# Foucault and Hayek on public health and the road to serfdom

**DOI:** 10.1007/s11127-021-00926-6

**Published:** 2021-09-07

**Authors:** Mark Pennington

**Affiliations:** Department of Political Economy, King’s College, University of London, London, UK

**Keywords:** Foucault, Hayek, Public health, Social constructionism, Narrative political economy, B00, B50, B53, B55

## Abstract

This paper draws on the work of Michel Foucault and Friedrich Hayek to understand threats to personal and enterprise freedom, arising from public health governance. Whereas public choice theory examines the incentives these institutions provide to agents, the analysis here understands those incentives as framed by discursive social constructions that affect the identity, power, and positionality of different actors. It shows how overlapping discourses of scientific rationalism may generate a ‘road to serfdom’ narrowing freedom of action and expression across an expanding terrain. As such, the paper contributes to the growing literature emphasising the importance of narratives, stories and metaphors as shaping political economic action in ways feeding through to outcomes and institutions.

## Introduction

The coronavirus pandemic has led many countries to introduce unprecedented restrictions on personal and enterprise freedom, restrictions justified by scientific discourses on ‘public health’. Prior to the pandemic those discourses appear also to have contributed to the expansion of the regulatory state into ‘lifestyle governance’. While not rejecting a role for the state in public health, this paper draws on the perspectives of the French social theorist Michel Foucault and the Austrian economist Friedrich Hayek to illustrate the mechanisms through which public health discourses may undermine a liberal social order. Whereas public choice theory focuses on the incentives facing agents to examine related questions, this paper understands those incentives as shaped by discursive social constructions, and especially a scientific rationalism that may affect the identity, power, and positionality of the actors concerned.

Foucault’s perspective has been very influential for interpretive political scientists (Bevir & Blakely, 2018) and the analysis here shows its relevance for the ‘economic approach to politics’. Hayek’s perspective is better known to economists and his ‘road to serfdom’ thesis shares important parallels with public choice accounts (Boettke, 1995). The similarities between Hayek’s subjectivist socioeconomic theory and Foucault’s social constructionist perspective may, however, be less familiar to *Public Choice* readers, much as they may be to Foucauldian analysts who often consider liberal political economy guilty of atomism and reductionism. What follows, therefore, is a provisional attempt to highlight some empirical and normative issues that bridge the political economy focus of *Public Choice* with the social constructionist elements in Foucault’s and Hayek’s work.

In making the connections the paper contributes to the Hayekian, Foucauldian, and public choice literatures related to public health. First, it adds to the small but growing Hayekian literature on the ‘knowledge problems’ of pandemic response (Bylund & Packard, 2021; Candela & Jacobsen, 2021; Coyne et al., 2021; Pennington, 2021; Storr et al., 2021). Whereas those contributions emphasize problems of uncertainty in identifying appropriate policy, this paper focuses on how background discursive constructions may empower certain actors working in the same epistemic conditions to define ‘solutions’ and to ‘police the truth’. Correspondingly, the paper complements the Foucauldian literature on ‘bio-power’ and ‘power-knowledge’ that emphasises the role of scientific rhetoric in sustaining the prestige of public health experts (Esposito, 2011; Hannah et al., 2020). Hayek’s critique of ‘scientism’ will be shown to complement the Foucauldian approach by destabilising the epistemic authority of such expertise.

With respect to public choice theory, significant work emphasizes the importance of rent seeking and bureaucratic capture in public health policies (Leeson & Thomson, 2021) and in related fields such as disaster management (Shughart, 2006). Though not yet applied to public health a literature emphasising how discursive–cultural phenomena may impact rent-seeking dynamics is growing. Choi and Storr (2019), for example, show how rent seeking can be activated within cultural–discursive settings that socially construct political activity in zero sum or negative sum terms. Elsewhere, Chamlee-Wright and Storr (2010, 2011) explore the role governmental narratives, and the stories citizens tell about their governments can play in affecting the expectations and abilities of communities to address collective action dilemmas and to limit rent seeking. This paper adds to that literature by exploring how public health discourses may marginalise certain subjectivities through a moralization and essentialization of externalities and collective action problems. It also points to the role played by discourse in structuring the capacities of different groups to engage in successful rent seeking.

The paper commences by synthesizing Foucault and Hayek on the political economy of freedom, social constructionism, and social control**.** It proceeds to analyze the processes through which liberal freedoms might be undermined in the context of pandemic response. It turns finally to broader discursive processes that predate the current pandemic and that may already have been morphing ‘constitutions of liberty’ into ‘constitutions of control’.

## Foucault and Hayek on self, social constructionism, and social control

Michel Foucault and Friedrich Hayek are two of the most influential social theorists of the last century. While Foucault often is associated with the radical left and Hayek with ‘neo-liberalism’, their respective social theories draw on common concerns offering a powerful cultural-economic lens through which to approach the political economy of public health.

### Foucault and Hayek on self, social constructionism, and agency

At the core of Foucault’s work is an anti-essentialist or ‘decentered’ conception of the human subject. In contrast to rational choice theories that posit the primacy of an autonomous agent with an underlying nature, Foucault sees the self as an unstable property emergent from the zone of conflict between different ‘discourses’ that frame how people interpret the world and their places within it. On that view, individual identity or subjectivity does not accord with underlying essences but is ‘imprinted’ on human subjects by the discursive modes to which they are exposed.

Closely allied is Foucault’s understanding of power. While not ignoring juridical powers centered in the state, or power that reflects the bargaining position of particular interest groups as emphasized in public choice analyses, Foucault offers a ‘decentered’ analysis wherein no identifiable ‘inventor’, institution, or place exists to which power can be traced. The ‘discourses’ that structure people’s patterns of thought emerge through multiple and dispersed attempts by actors to assert their places in society and to exert influence over others through an epistemic variant of Nietzsche’s ‘will-to-power’. That power ‘circulates’ or ‘flows’ through people and is modified by them in strategic ‘power-knowledge’ games. While such games involve some goal-oriented action, individuals are not always consciously aware how their actions contribute to the emergence of a wider set of norms that constrain and facilitate the actions of others. For Foucault, discursive power is an emergent property that has no ‘author’ and cannot be attributed to discrete intentional acts or to the control of resources and positions of authority (see, for example, Foucault, 1980, part 4).

Although his account sometimes is represented as one denying agency, Foucault (1982) was clear to emphasize that people can *choose* to submit to the identity discursive constructions create for them, or they can choose to *resist* the constraints they impose on them, ‘destabilising’ prevailing discourses by exploiting gaps or inconsistencies within them. Members of marginalised subjectivities such as gays have, for example, challenged stereotypes of homosexual ‘effeminacy’ by adopting ‘masculine’ comportments or have adopted scientistic discourses that depicted same-sex practices as ‘deviant’ to seek recognition for a natural basis to homosexuality. And as same-sex partnerships have moved out of the shadows, they have made visible to ‘heterosexuals’ the possibility of sexualities that cross classificatory boundaries. Such agency, and the social reflexivity it engenders may, however, be blocked whenever the normalizing effects of discourses align or crystallise with each other and with the juridical authority of the state. Thus, in a society where same-sex practices are not merely marginalised by dominant discourses but are *outlawed*, then mobilizing resistance against those discourses may be that much harder.

While the economic liberalism associated with many public choice theorists often is accused of ‘atomism’, Hayek’s economic liberalism shares with Foucault a thoroughly socialized conception of the human subject. Whereas Foucault sees persons as situated and individuated within ‘discourses’, Hayek (1948) sees persons as cognitively limited agents situated within multiple ‘streams of tradition’. The habits and traditions constituting personal identity are emergent social constructions that coordinate individual actions. Those constructions are not ‘natural’, but neither are they the result of deliberate ‘invention’ or ‘authorship’. Language, for example, develops as an unintended consequence of multiple communicative acts. As new words, combinations and meanings spread by a process of imitation their initiators are not consciously aware how they will be used and adapted by others (Reksulak et al., 2004). For Hayek then, as for Foucault, the social construction of reality is a *non-reductionist* process of ‘emergent’ or ‘spontaneous order’ that amounts to far more than an aggregation of individual decisions.

Where Foucault sees individuals as shaped, though *not determined* by discursive power formations, Hayek (1952) views the human mind as a classification system that interprets the external world through culturally acquired rules. People rely on traditions they find ‘given’ in the social environment and that seem successful for neighbouring agents, to cope with uncertainty. Creative agency, meanwhile, can be exercised as individuals ‘play’ with the culturally acquired classifications that organize thought, and in the process evolve new patterns of meaning and knowledge. As with Foucault, agents can *choose* whether to be ‘docile bodies’ accepting the traditions in which they are embedded, or they can *challenge* aspects of these traditions. Those willing to face opprobrium from challenging a social practice or construction may reveal to others new and potentially better modes of conduct. Knowledge of such potentialities is dispersed widely across society and could never be perceived in their entirety by any one group. For Hayek, that is a primary reason to favor traditions and informal norms over the formalized regulations issued by states. Informal rules may be easier to resist at the local level while changes to formal rules may require large-scale collective action and may draw only on the limited imagination of their designers (Hayek, 1958).

### Foucault and Hayek on subjectivity, science, and social control

Within his account of discursive power, Foucault draws attention to ‘disciplinary’ power and ‘bio-power’, which he associates with scientific discourses that attempt to categorize people and to generate knowledge about their behavior by applying quantitative techniques. Disciplinary power works by normalizing moralistic judgments classifying individuals in relation to norms then used as the basis for ‘corrective’ measures (Foucault, 1977). Although ‘discipline’ is more extreme where ‘deviance’ is greatest—as with imprisonment for criminals—milder disciplines operate in family life, educational establishments, doctors’ surgeries, military barracks, factories, the media, and public agencies—all of which act as decentralized sites for the evaluation and correction of behavior. Where the ‘disciplines’ focus on individuals, bio-power operates through social technologies that target social, economic, or environmental conditions as the objects of intervention (Foucault, 2007/1977, 2008/1978–1979). Such discursive power interacts with disciplinary power through ‘security’ mechanisms justified in the name of ‘population health’. Attempts to control sexual practices, for example, might overlap with concerns about the reproductive state of a population or the need to control the spread of disease.

In Foucault’s view, as people become the ‘objects’ of those scientific ‘gazes’—a process of ‘objectivation’—they may *acquire* a subjectivity or sense of individuality—a process of ‘subjectivation’. Thus, while same-sex practices had existed throughout time, the notion of ‘the homosexual’ as a deviant ‘type’ requiring ‘correction’ arose only with the scientific disciplines of sexology and psychiatry (Foucault, 1980). In turn, as people engaging in same-sex practices became the ‘object’ of scientific knowledge, a subjectivity experienced and internalised by many people as ‘homosexual’ was *activated*.

It is important to emphasize that Foucault does not see the effects of such scientific ‘power-knowledge’ in exclusively positive or negative terms. Scientific discourses can produce subjects capable of achieving outcomes (athletic bodies, high productivity, and healthy environments) that might not otherwise be possible. Equally, however, those discourses may limit freedom and reflexivity when they present as ‘totalizing’ or objective truths, often partial and subjective perspectives restricting what can be said and done—and especially so when intertwined with the juridical power of the state. Scientific authority is both reflective of the power conferred on those identified as ‘experts’ by social discourses, and an effect of the actions of scientists in seeking to influence those discourses. Since no underlying human nature or essence exists, positivist notions of law-like regularities in human behavior that can be uncovered by science need to be ‘destabilised’. That analysis does not reject enlightenment values per se but suggests that they represent a *particular* form of rationality that, as Kuhn (1962) also emphasised, may reflect power-relationships influencing what is considered true, *irrespective of whether it is true* (Foucault, 1997). Sustaining freedom and reflexivity, therefore, requires institutions that allow dissensus between *plural* rationalities (Lemke, 2012, chapter 4).

Where Foucault emphasises the importance of a *pluralism of rationalities*, Hayek (1957, p. 52) argues that,

It is probably no exaggeration to say that every important advance in economic theory during the last hundred years was a further step in the consistent application of *subjectivism.*

That subjectivism refers not only to preferences, but to varied interpretations of economic conditions, as well as expectations about future states of the world. Economic ‘data’ are social constructions that may be interpreted differently by people with different cognitive frames, or by the same person changing their construction of the same ‘data’ at a *later* point. Where Foucault destabilizes behavioral regularities associated with fixed-identity types in the human sciences, Hayek destabilizes the static equilibrium relationships between economic variables in positivist economic theory. In a market economy based on decentralized though unequal property ownership and with open competition, knowledge of ‘more’ or ‘less’ cost-effective actions evolves *inter-subjectively* from the clash of varied and overlapping interpretations of economic conditions. As with traditions, the market prices emergent from such processes provide *some* regularities that people can use to navigate an uncertain world, but those regularities will be temporary. That conclusion follows because while entrepreneurs may react passively to current prices, others may challenge or ‘resist’ them by bringing forth new ideas that ‘strike at the foundations and very lives of incumbent firms’ (Schumpeter, 1942, p. 84). Nor are consumers passive agents who necessarily accept the goods targeted at them by company advertising—they may creatively challenge entrepreneurial expectations by rearranging their consumption plans in entirely unexpected ways.

On a Hayekian view, those dynamic and reflexive processes may be suppressed by scientistic attempts to extend the capacity for prediction and control that may be appropriate in the natural sciences or in the analysis of ‘simple’ phenomena, to the manipulation of complex socioeconomic relationships. It is a ‘pretense of knowledge’ or, in Foucauldian terms, a totalizing ‘power-knowledge’ claim, to suggest that the generation and interpretive processing of the relevant data can be centralized in the hands of an organization or group to plan economic activity, or to ‘correct’ the ‘messy’ reality of competitive market processes (Hayek, 1978).

Within that context, Hayek’s *Road to Serfdom* thesis explains how attempts to introduce ‘scientific social planning’ in the absence of an objective social welfare function may mutate into more, or less brutal power struggles between rival interests, each seeking to impose its own partial and subjective perspective on the whole (Hayek, 1944/1972). Although his thesis often is misrepresented by critics for depicting a stark choice between freedom under laissez faire or a state of authoritarian unfreedom flowing from *any* attempt to intervene in private markets, Hayek supported an extensive range of government services, including public health functions, that he believed to be compatible with a liberal order (Ibid.; Hayek, 1960). What his ‘serfdom’ thesis suggests is that *beyond a certain point* the entanglements between politics and markets may mutate to transform a ‘constitution of liberty’ into a ‘constitution of control’ (Wagner, 2016).

### Synthesis

It should now be apparent that Foucault and Hayek share an anti-essentialist account of the human subject, a social constructionist epistemology, and a focus on non-reductionist processes (discursive power relations for Foucault; traditions, language, and prices for Hayek) wherein social order is understood not in terms of the intentions of specific individuals or groups but as arising unintentionally from ‘decentered’ or spontaneous ordering processes. Foucault and Hayek also share a fluid or ‘disequilibrium’ understanding of social ‘order’ and an appreciation of the threats to that fluidity arising from scientistic modes of thought that seek to standardise behavior and especially so when institutionalised in the apparatus of the state.

While their similarities should be clear, the analyses presented by Foucault and Hayek each contain insights and emphases neglected by the other. Notably, Foucault’s focus on cultural–political phenomena leaves the economic implications of the plural-rationalities perspective underexplored. Hayek’s critique of socioeconomic ‘scientism’ therefore, offers an opportunity to complement the Foucauldian ‘power-knowledge’ framework. Such cultural-economic synthesis may particularly be important for, as Hayek notes, no purely economic ends exist—‘economic’ decisions are linked inherently to the conflicting tradeoffs between diverse cultural values in any pluralistic social order—a pluralism threatened by attempts to integrate those values into a single ethical code (Hayek, 1944/1972, p. 61).

In the case of Hayek, while his ‘serfdom’ thesis explains how excessive attempts to replace markets with state planning may undermine liberal freedoms, he does not analyze explicitly the factors influencing the interests that might prevail in such struggles. A public choice explanation would examine the incentives for different actors to mobilize collectively and to capture the state apparatus (Boettke, 1995). Foucault’s perspective, however, emphasizes that actors’ perceptions of their interests and the incentives they face will be conditioned by prevailing discursive constructions. ‘Objectivation’ and ‘subjectivation’ processes may affect how actors define their interests and may be used as a strategic or communicative resource by which to advance them. By appreciating how discourses, narratives and world-views link and cross fertilize, how emergent ‘discursive formations’ work to define or construct relationships between different actors, and how they interact with juridical authority, one can attempt to discern the power relations the constructions may create and/or sustain. Crucially, the effect of such discursive constructions may conceal the operations of power. The ‘road to serfdom’ need not be strewn with bloodied or imprisoned bodies but may unfold through more or less imperceptible processes establishing ‘taken for granted’ assumptions that prevent the consideration of alternatives. Hayek himself refers to a ‘softer’ form of the ‘serfdom’ thesis not involving nakedly authoritarian institutions and Foucault’s account may help to explicate these very processes (Hayek, 1944/1972, preface to 1956 edition, p. xxxiv).

While it may seem alien to the public choice theorist, the importance of discursive constructions increasingly has been recognized by some influential economists. Shiller (2019), for example, examines the impact narratives have in shaping economic behavior. He argues that stories and metaphors influence people’s ‘loose thinking and actions’ and work as ‘contagions’ that become accepted so widely that they become one of the primary reasons for action that feeds through to economic outcomes and institutions. That focus on narrative likewise is evident in Denzau and North’s (1994) notion of ‘shared mental models’ and their power especially in non-market settings where it may be harder for agents to test the claims made for one institution or policy over another, than it is for people to test the claims made for commercial products. Such an approach avoids the functionalist explanations in some public choice analyses and in neo-Marxist accounts that interpret the discursive realm as a merely epiphenomenal effect of underlying or ‘real’ interests. On a Foucauldian–Hayekian view, prevailing discourses and traditions may emerge or become established through historical accidents, that may then work to shape or produce the interests of those situated within them. The discourses of scientific rationalism that will be the focus here, for example, were not necessarily ‘invented’ to serve the interests of scientists, but once established they may nonetheless shape those interests as well as of other actors and be used as a strategic resource to defend or advance them.

In what follows the Foucauldian–Hayekian framework will be deployed to analyze the political economy of public health. The latter involves regulatory interventions influenced heavily by scientistic discourses that cut across cultural identity and economic decision-making. Public health also is a field wherein linkages may be discerned between natural and social scientific discourses that legitimate forms of governance that may reduce freedom of expression and action. In exploring these possibilities the strategy deployed here is not to examine in close empirical detail the contours of the relevant discourses and associated policy responses, but rather to highlight broadly discernible patterns that explicate the potential value of the Foucauldian–Hayekian approach. As such, the paper aims to stimulate a new stream of political economy research that may generate more fine-grained empirical work in due course.

## Public health and the road to serfdom: the problem of infectious diseases

Public health is an expansive concept. According to Winlsow’s (1920, p. 23) classic definition,

Public health is the science and art of preventing disease, prolonging life and promoting physical health and efficiency through organised community efforts for the sanitation of the environment, the control of community infections, the education of the individual in principles of personal hygiene, the organisation of medical and nursing services for the early diagnosis and preventive treatment of disease and the development of social machinery which will ensure to every individual in the community a standard of living adequate for the maintenance of health.

Within that context, two discourses that appear to have structured responses to the coronavirus pandemic and the ‘lifestyle governance’ policies predating it, will be the focus here. The first such discourse originates in epidemiology. Here the human body is understood as a vector for contagion with policy focused on regulatory interventions that can control the spread of diseases, including quarantines and other ‘social distancing’ measures, as well as possible requirements for vaccination to achieve ‘herd immunity’. A parallel concern relates to preventive measures such as improved personal hygiene and better sanitary conditions (such as clean water supplies and effective sewerage systems) that reduce the prevalence of disease.

This epidemiological discourse has close affinity with the economic discourse of modern neo-classical welfare theory and its analysis of externalities and collective action problems that may inhibit optimal outcomes. In the context of infectious diseases, individuals not social distancing and taking risks with their health are understood to ‘impose costs’ analogous to a form of ‘pollution’ on innocent parties. While externalities might be dealt with through private bargaining or by taxing the activities concerned, the high transaction costs of such methods in emergencies may justify ‘command and control’ measures, a logic also applied to arguments for compulsory vaccination to eliminate ‘free-riding’. The same logic likewise may justify publicly supplied and financed sanitary infrastructures when collective action problems may block decentralised supply.

This paper does not deny that the situational logics analyzed by epidemiologists and welfare economists *may* lead to unsatisfactory outcomes; neither does it view public health measures as *necessarily* representing a ‘road to serfdom’. Nonetheless, on a Foucauldian–Hayekian view apparently neutral and scientific discourses may conceal subjective judgments and power-knowledge claims that moralize and essentialize understandings of both individual and collective problems in a manner pregnant with threats to freedom of action and expression. The remainder of this section considers those threats in the context of pandemic response. The subsequent section examines the wider import of those discourses on public policies pre-dating the pandemic.

### Erasing complexity and uncertainty

The first threat to consider arises from the social construction of policymakers as objective data analysts who identify and correct disequilibria by selecting the least-cost option(s) that generate the highest value in terms of improved health—or some combination of health and other goals. In the context of the coronavirus pandemic, while it is acknowledged widely that policy has been made in a context of limited information, what Foucault refers to as ‘bio-political’ discourses nonetheless may have framed the issue as one wherein the appropriate policy response is accessible to epidemiologists and social scientists. A Foucauldian–Hayekian perspective would, however, seek to ‘destabilize’ that position. For Hayek in particular, it is not simply a case of waiting for ‘more data to come in’, since the quality of those data will depend on the nature of the social process through which it is *produced and constructed*. Just as central economic planning deprives consumers and producers of data about possible opportunity costs generated by competitive experimentation in a market, so any *singular* approach to the governance of a pandemic may reduce the generation of counterfactuals and alternative problem constructions.

Moreover, even if produced by a pluralistic process, ‘the data’ will not ‘speak for themselves’. If infectious diseases are complex phenomena, then they may exhibit *unpredictable* responses to differences in geography, weather, and public policy interventions. Equally, if the cultural-economic systems wherein policy makers and diseases intervene are themselves complex phenomena then differences in economic, cultural, and institutional circumstances may generate considerable unpredictability about the effectiveness of alternative policy regimes (Bylund & Packard, 2021; Coyne et al., 2021).

In such circumstances, the data necessary for identifying and correcting socioeconomic disequilibria will be subjective, uncertain and their robustness dependent on a reflexive process wherein different social constructions continually can be tested against one another. In the context of the coronavirus pandemic, the fragmented nature of governance arrangements across the world, combined with the freedom of expression routinely exercised under liberal and social democratic governments, has sustained *some* level of pluralism and reflexivity. Nonetheless, the ways in which such diversity has been greeted point to the possible ways that discourses of scientific rationalism may undermine that very pluralism.

First, from a Foucauldian perspective in a discursive context wherein the social status of public health professionals may be derived from their perceived ability to provide ‘scientific answers’, few professional rewards may be available from emphasizing uncertainty and demonstrating an unwillingness to offer a ‘solution’. The interactions of public health professionals with politicians eager to secure support by presenting themselves as having solutions, and media outlets keen to secure audiences by presenting those answers to the population, may frame an incentive structure or selection effect favoring those downplaying uncertainty. That may be an especially important dynamic in a discursive setting with expectations that emergency situations such as pandemics require that ‘something must be done’—and especially by governments.

Second, when discourses of scientific rationalism provide social status for public health professionals, their identity may be threatened should ‘too many’ conflicting views be raised. When the public and media ‘gaze’ is focused on professional claims to provide ‘answers’, a perceived inability to do so may escalate scepticism of public health expertise in other domains. Within this context, Koppl (2018, chapters 3, 9, and 10) notes that many professional bodies routinely limit diversity of opinion based on fears that the public appearance of ‘too much’ disagreement will undermine the credibility of professional expertise. Disciplinary pressures thus may be brought to bear on dissenters. In the context of the current pandemic notable attempts have been made by public health scientists, actors in civil society and the private sector, and especially social media platforms to ‘police’ the parameters of ‘the science’. Economists have been less visible in public debate, but publicly visible opinion also shows signs of pressure to conformity, with expressions of support for government policies early in the pandemic (*Financial Times*, April 4th, 2020), notwithstanding a lack of available data and the fact that welfare economics offers no clear guidelines on the wisdom of the policies adopted (Boettke & Powell, 2021).

Setting aside removal of conspiracy theorists, the opinions of epidemiologists and economists that challenge the dominant narrative in favor of ‘lockdowns’ or radical social distancing regimes have seen their circulation restricted. Social media channels such as You-tube have exercised disciplinary power, banning or placing ‘warnings’ on advice that goes against World Health Organisation guidelines or those of local public health authorities (see for example, *Wall Street Journal*, December 7th, 2020*; Washington Post*, September 17th, 2020). Neither have those pressures been confined to individuals—countries such as Sweden and, in the United States, jurisdictions such as South Dakota and Florida routinely have been condemned for not following the dominant narrative favoring extreme social distancing.

Those Foucauldian disciplinary dynamics have important parallels with Hayek’s ‘serfdom’ thesis—when faced with the inability for state planners to agree on the contents of a unified plan, pressure mounts for a ‘strong man’ to ‘take charge’ and to impose a particular ‘will’. In the case of pandemic response, the equivalent figures providing the necessary coherence to professional opinion may not be authoritarian ‘strong men’ but the leading public health officials in government agencies such as the Center for Disease Control (CDC) in the United States and Scientific Advisory Group for Emergencies (SAGE) in the United Kingdom. Those officials have the public and media platforms to specify what ‘the science’ says, a message that may then be enforced by the actions and inactions of agents within the relevant professional networks, as well as by politicians, the media and civil society. Such actions may not necessarily be driven by ‘the science’ but may be affected by the flow of events and how they are discursively constructed by actors in society at large.

It is important to emphasize here that the operation of such discursive power is not necessarily a ‘top-down’ phenomena but may arise through an interaction effect when the options available to top-down actors such as CDC or SAGE officials are structured by a combination of events, institutions, and the discourses circulating in society. In the specific case of the CoVid19 pandemic, preparedness advice from bodies such as the World Health Organisation and SAGE (WHO, 2019; UK Government, 2011) was that ‘lockdown’ measures were undesirable or ineffective and the initial response in countries such as the United Kingdom stuck to that line.^1^ It was only *after* the Italian and then the Spanish administrations imitated the Chinese ‘lockdown’ response, soon followed by others, and the subsequent clamor from many parts of civil society and the media that policy changed—and was then presented as that required by ‘the science’ (for a content analysis of Covid19 media framing, see Ogbodo et al., 2020). In this instance, pressure towards convergence on ‘lockdowns’ was driven partially by bottom-up forces and it may have been the wider institutional separation of the public health bureaucracy in Sweden from the media and political pressure that accounts in part for its *relative* insulation from such forces. In the latter instance, pluralism was limited from the top down, as officials coordinated on a strategy that *rejected* lockdowns, as *not* supported by ‘the science’—notwithstanding pressures from some Swedish localities for implementing such measures (Bylund & Packard, 2021).

Third, once official opinion has aligned, dissenters from the publicly dominant scientific narrative may be criticized for corruption or ‘deviance’. In the case of the Covid19 response, that tendency may accord with the often highly certain and moralistic tone adopted in debates surrounding the appropriateness of ‘lockdown’ regimes with some opinions and policies described as ‘following *the* science’ and others deemed as non-scientific, immoral, or corrupt**.** That combination of epistemic certainty and moralism has not, it should be emphasized, been confined to supporters of radical social distancing. The signatories to the Great Barrington Declaration which opposed lockdowns favoring instead ‘focused protection’ have been equally confident in their pronouncements. That ‘lockdown sceptics’ have been subject to attempted censorship and disciplinary social pressure may merely be reflective of their minority standing in a context where objective truths are assumed accessible to experts.

While they have for the most part been sustained, the various attempts to ‘police the truth’ suggest that pressures generated within discourses of scientific rationalism may exercise a chilling effect on both freedom of expression and of action.

### Moralizing interpersonal conflict

A second set of threats to a liberal order may arise from the strategic deployment of contagion discourses in epidemiology and externality discourses in welfare economics. As Coase (1960) argues, the problem of ‘imposed costs’ occurs whenever *conflicting* subjective evaluations of risks arise, and when a need exists to balance or adjudicate the conflicting interests and/or points of view. In the context of infectious diseases, people reluctant to social distance may harm others, especially those with underlying health conditions. Nonetheless, people wanting to eradicate infections likewise may harm those with vulnerabilities to economic or other forms of distress arising from ‘excessive’ public health concern.

In neoclassical economic discourse, the focus is on identifying Pareto-relevant externalities, measuring them, and devising mechanisms that will internalize costs—activities that it is assumed can and will be carried out in a neutral manner by professional economists, politicians or judges acting separately or in concert. Hayek’s perspective, however, questions the capacity of humans to generate and to process the relevant ‘data’ in a neutral and objective manner. When little or no objective data may be available to assess the sizes or directions of externalities or whether private actions are sufficient to internalise them (on such actions, see Leeson & Rouanet, 2021) then Foucauldian ‘power-knowledge’ claims may assume considerable significance with ‘externalities’ used as a rhetorical device to seek power over other actors or to redistribute rights and resources through what public choice theory would describe as rent seeking actions. Grow Sun and Daniels (2016) refer here to the important role played by ‘externality entrepreneurs'. Such entrepreneurs seek discursively to frame and construct whom to *brand* as the moral *wrongdoer*, depending on where they believe the greatest reservoir of support in the media, civil society and politics may lie, or where it might be generated. Discursive entrepreneurship may be crucial in communicative contexts wherein media agencies may have commercial imperatives to cover news items that can be depicted in highly dramatized forms (Rydin & Pennington, 2001; Yates & Stroup, 2000).

Consequently, instead of viewing the management of disease as an interpersonal conflict between subjects who perceive risk in different ways, contagion and externalities’ narratives may enable a *moralization of* distinctions between actors labelled ‘perpetrators’ of harm and those described as ‘victims'. When such moralistic certainties are mobilized by public and private agencies to ‘discipline’ and ‘correct’ perpetrators then the ‘objectivation’ process may activate a subjective experience of ‘moral deviance’ for those concerned. Such tendencies appear to have been evident in political and media discussions of Covid19 responses where citizens resisting ‘lockdown’ regimes have been demonized in public and media commentary as ‘selfish’, ‘ignorant’ or ‘lacking in community spirt’, (see, for example, comments by the UK Secretary of State for Health, *The Guardian*, March 23rd, 2020), as well as being subject to fines and a complex apparatus of public and private surveillance (Coyne & Yatsyshina, 2020; Hannah et al., 2020).

Now, of course, some diseases may pose such enormous threats to life and health that only a tiny minority would view restrictions in a negative light, and when it would be far-fetched to consider them to breach pluralistic standards. The problem here, however, is that such clear-cut cases may be the exception rather than the rule. With Covid19, it is far from self-evident that health risks even to the most vulnerable elderly people, significant though they may be, outweigh the poor mental health, suicides and lost family interactions inflicted by radical social distancing. From a plural rationalities or subjectivist perspective, it may be no less reasonable for an 80-yearold woman to want to take their chances and to continue seeing loved ones, than it may be for a similarly situated person to wish for themselves and others to self-isolate.

The point here is that decisions to favor one side or another in such conflicts are not necessarily ‘wrong’ or ‘inefficient’ but that they may generate or reflect biases that *limit pluralism*. Graphic presentations by politicians and the media of people dying in hospital beds combined with contagion narratives have, for example, been exploited in many countries to galvanize support for lockdown measures when many conditions that subsequently have gone untreated, and the severe mental health challenges that may follow such measures, may be less amenable to such imagery. The threat to pluralism here may be most pronounced in unitary governance structures that impose a single ‘solution’, but even in more fractured governance regimes where ‘different sides’ may be taken in different places (on the case for them, see Congleton, 2021), contagion narratives, the pressures towards conformity of professional and public opinion discussed previously, and moral certainties about who is in the ‘wrong’, may overwhelm that pluralism.

### Essentializing people and essentializing collective goods

A third threat to pluralism may follow from the *essentialization* of agents facing public health problems, such as pandemic response, and the structural features of the relevant issues. Specifically, the focus on contagions that demands social distancing, and a rational choice discourse depicting agents as narrowly self-interested and prone to free-riding, constructs an inexorable logic wherein intervention by central government agencies *must* be employed. On a Foucauldian–Hayekian view, however, by so constructing those problems, the cultural heterogeneity and the plural or subjective rationality of the relevant agents, may be neglected, with differential individual and cultural propensities to free ride or to perceive issues as having a collective goods character, erased from view.

While it does not rule out scenarios wherein collective goods would be supplied insufficiently by decentralized provision mechanisms, a plural rationalities or subjectivist perspective destabilizes essentialist understandings suggesting that very few goods may exist that *must* be supplied in a particular format. Whether a good has collective good properties and whether those characteristics compromise the viability and efficiency of a pluralist model may be *culturally contingent*. In the context of pandemics, it may be that the governance challenge does not imply a *singular* collective action problem but involves a *plurality* of localized collective action problems, the structures of which may vary according to the cultural characteristics and beliefs of the populations concerned. In a manner commensurate with Foucauldian genealogy, political economists in the Hayekian, Coasian, and Ostromian traditions have exposed numerous historical cases of alleged collective action dilemmas—from the supply of lighthouses to television signals and from sewerage systems to the provision of streetlights—which far from displaying an underlying ‘essence’, creative entrepreneurship has enabled actors to supply without recourse to centralized governmental action (Spulber, 2001).

Cast in Foucauldian–Hayekian terms, the apparent unwillingness of public bureaucracies, public health experts, much of the media and public opinion to countenance pluralism may be reflective of dominant discursive constructions that privilege public regulators and that denude the potential for more localized agency. On the one hand, the contagion and externality discourse may provide ‘externality entrepreneurs’ in public health bureaucracies and interest groups with a communicative resource to secure power and money at the expense of other actors. On the other hand, the institutionalization of discourses within public organisations and the media that depict the structural properties of infectious diseases as large-scale collective action dilemmas, reinforced by the circulation of emergency/contagion narratives, may work to *produce* a subjectivity of helplessness on the part of many citizens—a situation wherein ‘self-governance’ is experienced as a practical impossibility. That argument does not imply that satisfactory or efficient pluralistic solutions to pandemics in fact exist—but is to suggest in a Foucauldian vein that dominant discourses may work to influence what is considered to be true, *irrespective of whether it is true*. Prevailing public health narratives may prevent individuals and communities from discovering whether they might craft satisfactory responses without recourse to centrally imposed solutions.

## Public health and the road to serfdom: the viral *dispositif*

The previous section highlighted how political economic pluralism might be threatened by scientistic discourses on public health. However, reasons can be found for treating the ‘road to serfdom’ thesis with circumspection because the potentially emergency nature of pandemics and the difficulty of allowing plural forms of action to co-exist also must be recognised. Foucault (1963/1973) notes that the spread of infectious diseases panicked political authorities in post-revolutionary France, inducing centralizing measures that reversed the previous trend of radical liberalisation. Neither should the efficiency of such measures be ruled out—it may be that all things considered once a decision is made to adopt emergency measures that simple public health messaging downplaying uncertainty and marginalizing dissenting voices is required to sustain support, as likewise might be the case during wartime.

Nonetheless, the speed with which liberal and social democratic societies have abandoned long-established public freedoms and have embraced controls, often more extensive than in wartime, may be reflective of the rising powers exercised by ‘bio-political’ discourses that *predate* the pandemic. Those discourses routinely justify disciplinary measures, albeit in milder form, that limit pluralism and ‘police the truth’. It is to explicate how the discursive context of public health governance already may have been morphing ‘constitutions of liberty’ into ‘constitutions of control’ prior to the pandemic, that the remaining pages now turn.

### The viral dispositif

Foucault (1977) adopts terms such as the ‘apparatus’ or ‘*dispositif’* to refer to an alignment between various discourses, institutions, administrative decisions, and scientific and philosophical statements that limit the construction of issues and that may narrow the scope for alternative narratives and understandings. Recent years may have witnessed the emergence of such a *dispositif*, combining epidemiological understandings of contagions and economic accounts of externalities and collective action dilemmas, across a raft of health-related issues. Indeed, it might be argued that the social construction of those issues in terms of epidemic or contagion effects has itself developed a contagious quality. The phenomena of people smoking (or switching to substitutes such as vaping), being above a certain weight, or consuming alcohol now routinely are constructed as effects of a form of *epidemic* with the relevant bodies seen as vectors for harmful behavioral practices (Anomaly, 2012; Cotter et al., 2021; Mitchell & McTigue, 2007).

As with infectious diseases the contagion/emergency narrative has overlapped with an economic discourse constructing the same behaviors as involving externality and collective action problems (Anomaly, 2012). The existence of what are deemed harmful consumption practices and the advertising that promotes them, frequently are portrayed as forms of ‘pollution’. With tobacco, for example, *any* instance of smoking in the company of non-family members is interpreted as *physical* invasion of other peoples’ spaces and in many countries correspondingly has been banned in public and in private spaces where people gather—measures that may soon be extended to practices such as vaping (European Union, 2021)**.** With fatty or sugary foods and alcohol the pollution is considered cultural in form—where the presence of people engaging in those behaviors or of companies advertising them is presented as a ‘temptation’ to ‘deviate’ from ‘healthy behavior’. This logic has been pursued not only in the context of discussions about *interpersonal* relations but, in the form of behavioral economics, has extended to include judgments about plural values *within* persons. Specifically, the threat presented by the current desires of people to consume ‘less healthful’ products is constructed as ‘imposing a cost’ on their ‘future selves’—a cost demanding correction by external regulators (Rizzo & Whitman, 2019).

While a more detailed discourse analysis of how the component narratives interact in different countries and policy domains than can be offered here is required, the core elements of a Foucauldian apparatus or *dispositif* may be discerned, sharing much in common with the elements evident in the response to the new coronavirus.

First, power-knowledge claims to scientific objectivity have been made in delimiting healthful practices, claims that erase ongoing disagreements within scientific communities. In the case of tobacco, for example, while the danger to smokers from personal consumption had been established for years, the existence or magnitude of the health threats to non-smokers from ‘passive smoking’ was *not* established when public health professionals aligned with activist groups to demand extensive public regulation of smoking in public and private spaces (Berridge, 2011).^2^

Second, moralizing distinctions have been made between actors judged as perpetrators of harms against others presented as victims such that toleration between differing subjective evaluations of the tradeoffs between health goals and non-health goals has been displaced by a judgmental approach. The notion that, for example, people might enjoy engaging in various ‘risky’ practices by their decisions to eat certain foods, to smoke or to vape, increasingly is ignored or treated as morally dangerous (on the marginalising effects for smokers, see Bell et al., 2010). Consequently, in the case of high fat and high sugar foods, some governments are now considering advertising bans and restrictions on where retailers can display those products in their stores (UK Government July, 2020). In the case of tobacco, meanwhile, regulation has moved far beyond requirements for health warnings on packaging to include constraints on, or the elimination of, most advertising. In the UK specifically, retailers must ensure that products are *hidden from direct view* to further discourage consumption. Combined with restrictions on smoking in public places, the position regarding freedom to consume tobacco is now not dissimilar to the legal situation that faced gay men under the Sexual Offences Act of 1967—legislation that permitted homosexual acts, but only between two consenting adults *in private*—lest their public visibility ‘corrupt’ young males into experimenting sexually with other men.

Third, essentializing claims are made about individuals and the structure of their choice architectures, framing them as large-scale collective action dilemmas that preclude voluntary measures. Those claims are made notwithstanding practical illustrations of more pluralistic alternatives that might allow the relevant costs to be internalized at a relatively decentralized level. In the case of smoking, for example, a Hayekian mosaic of competing voluntary governance measures was practiced by many employers, restaurants and drinking establishments that allowed people to select between more- or- less restrictive approaches to the interpersonal conflicts that surround the use of tobacco—but they have since been swept away by more centralized and disciplinary governance regimes that often ban smoking in *all* public places (Meadowcroft, 2011).

While the discursive formations and the processes of ‘objectivation’ and ‘subjectivation’ entailed cannot credibly be said to determine people’s identities; on the view advanced here, they may nonetheless threaten personal and enterprise freedom and especially so given their institutionalization within the public health apparatuses of many states and supra-national bodies. The same processes also may explain why resistance to the huge infringements of personal liberty in the wake of the Covid19 pandemic has been so weak and, indeed, why many people in liberal and social democratic countries have *demanded* restrictions that public health professionals did not previously consider as having any chance of securing widespread legitimacy.

To be clear, the suggestion here is not that those measures are *necessarily* ‘wrong’ or ‘inefficient’, or that people believe that smoking and fatty food consumption are infectious diseases. Rather, the argument is that in a discursive–cultural context wherein epidemic, pollution and emergency narratives are now commonplace, the possibility that individuals might exercise choice and self-governance over health-related matters may have increasingly become alien (for a related discussion, see Buchanan, 2005).

Neither is there any suggestion that *all* regulations pertaining to smoking or the advertizement of fatty and sugary foods represents a ‘road to serfdom’. Constraints on fraudulent claims are entirely consistent with a ‘constitution of liberty’, as are attempts to expose manipulation of scientific research by large businesses or other interested parties. It is, however, not unreasonable to suggest that government bans and directives that limit where adults can view products in retail outlets might epitomize a ‘constitution of control’.

### Experts, interest groups and the viral dispositif

Crucially, the ‘road to serfdom’ appears to have emerged in ‘decentered’ or ‘spontaneous order’ vein from the interaction of events with the strategic deployment of contagion, externality and emergency narratives by public health professionals, economists, activist groups, medical establishments, and the media. The cumulative effect of those agents situating the pursuit of local objectives within their own discursive frames and borrowing concepts derived from overlapping discourses, may have led to the emergence of a cultural–discursive formation producing an amoeba-like growth of ever more centralised and prescriptive government controls.

Berridge’s (2011) analysis of public health regulation in the United Kingdom is instructive. In the case of tobacco, she notes how in the 1980s and 1990s an overlapping discourse emerged from the campaigns of anti-smoking groups and a radical sub-group within the public health profession and associated government agencies that shifted the emphasis from providing better information and of imposing taxes to discourage consumption, to one focused on *eliminating* smoking through the direct management of public and private spaces. A key element of their success arose from anti-tobacco advocacy groups drawing on public memory of high-profile smog incidents in the post-war era, alongside a similar memory of public inoculation campaigns against disease, to sustain a narrative wherein tobacco smoking was seen as pollution, and figuratively if not literally as a social contagion. Such activities illustrate the phenomenon of ‘externality entrepreneurship’, with the respective media campaigns framing what ‘the science says’ and of using a perpetrators and victims’ narrative to mobilize support for a centralized public response.

Now, understanding policy trajectories in relation to the lobbying of interest groups, bureaucratic agencies and their interactions with politicians is the standard fare of public choice theory. The value of the account presented here, however, and especially the Foucauldian notion of a ‘discursive formation’, is twofold. First, it suggests that the bargaining powers of interest groups and bureaucratic actors may be constrained by their capacities to creatively position their demands and interests *within* discursive constructions *not* of their own making. The power of dominant discourses cannot be attributed to the actions of any one group or set of interests but is emergent from the complex interactions between events and multiple localized though overlapping discursive strategies.

Second, it suggests that discursive coalitions can be mobilized to shape incentives and to produce outcomes that might *not* be expected by standard public choice accounts. Logic of collective action models might, for example, predict that public health regulation will be dominated by relatively small groups facing the lowest transaction costs of association and with a concentrated stake in outcomes, at the expense of larger, more diffuse constituencies that find it harder to overcome free riding**.** Industry groups and public bureaucracies may capture the regulatory process, reducing competition to the detriment of consumer welfare, while benefitting incumbent business profits and the budgets of regulatory agencies (Leeson & Thomson, 2021).

While not discounting the possibility of such regulatory capture, the Foucauldian perspective may help to explain a process of *ideational capture* whereby the deployment of contagion, pollution and emergency narratives circulating in society enables relatively small numbers of activist campaigners and public health professionals to mobilize public opinion through the media, in favor of centralized and moralistic controls. The strategic deployment of such discourses may account for the tendency evident with passive smoking, vaping and increasingly of food regulation for public health agencies to downplay uncertainty and to define ‘the science’ in a manner paving the way for top-down regulation. The deployment of those social constructions likewise may account for the apparent ‘defeats’ inflicted on industry interests by public health campaigners. Those outcomes might be explained by the power of contagion and emergency narratives to mobilize politicians into regulating private enterprise in a way that neutralizes or overcomes the organizational advantages that large business interests may have in overcoming collective action problems.

Those outcomes may not, however, represent a victory for consumers or for pluralism. On the contrary, from a plural rationalities perspective they may involve processes through which certain understandings of consumer interests are privileged while others are marginalised. Specifically, while consumers are portrayed in current discourse as the victims of corporate marketing, in cases such as smoking and food regulation they simultaneously are constructed as *perpetrators* of harms to others, or to themselves. Whereas public choice theory would suggest that large and diffuse consumer interests already may face severe collective action problems, this type of discursive framing may further *disincentivize* consumer mobilization. Pluralism thus may be stifled as the notion of a legitimate consumer interest in accessing tobacco, tobacco substitutes or certain foods at affordable prices, is erased from public view.

## Conclusion

This paper has offered an account of how discourses of public health governance may transform a ‘constitution of liberty’ into a ‘constitution of control’. While the processes of centralization and suppression of differences of opinion described herein might be seen as features of *any* government action, the account suggests that those tendencies are accentuated and given form by overlapping discourses of scientific rationalism. One implication of the analysis, therefore, is that pluralism and differences might be preserved should discourses that emphasize the limits of scientific reason achieve greater public currency.

The analysis is highly provisional and future work may examine in greater empirical detail than has been possible here how the discursive construction of ‘public health’ shapes incentives for individual and collective action or inaction across a range of domains. The path forward could include detailed histories, comparative case analyses or ethnographic studies. Alternatively, it may involve quantitative methodologies that look for statistical associations between the prevalence of certain narratives and discursive tropes and the regulatory measures adopted. Whichever methodology is deployed, any regularities unearthed should *not* be interpreted as empirical laws. The aim here has not been to suggest that ‘roads to serfdom’ are an inevitable consequence of scientific discourses of public health. The combination of the Foucauldian–Hayekian narrative with public choice analyses may offer the building blocks for a counter–discursive challenge that destabilises the social constructions that threaten liberal freedoms and that vaccinates liberalism against those threats.

## Footnotes

1 The reports refer to pandemic influenza and may not be thought relevant to Covid 19. Crucially, however, lockdown measures are advised against for scenarios with 15-week death rates between 200,000 and 300,000 (UK Government, 2011). No country, irrespective of policy, has experienced such death rates from Covid19.

2 Evidence of the negative health effects of passive smoking, as opposed to its nuisance value, continues to be inconclusive—see, for example, Ho et al. (2016) and Peres (2013).
